# Functions of p38 MAP Kinases in the Central Nervous System

**DOI:** 10.3389/fnmol.2020.570586

**Published:** 2020-09-08

**Authors:** Prita R. Asih, Emmanuel Prikas, Kristie Stefanoska, Amanda R. P. Tan, Holly I. Ahel, Arne Ittner

**Affiliations:** Dementia Research Centre, Faculty of Medicine, Health and Human Sciences, Macquarie University, Sydney, NSW, Australia

**Keywords:** signal transduction, central nervous system, neuron, astrocyte, microglia, oligodendrocyte, mitogen activated protein (MAP) kinase p38

## Abstract

Mitogen-activated protein (MAP) kinases are a central component in signaling networks in a multitude of mammalian cell types. This review covers recent advances on specific functions of p38 MAP kinases in cells of the central nervous system. Unique and specific functions of the four mammalian p38 kinases are found in all major cell types in the brain. Mechanisms of p38 activation and downstream phosphorylation substrates in these different contexts are outlined and how they contribute to functions of p38 in physiological and under disease conditions. Results in different model organisms demonstrated that p38 kinases are involved in cognitive functions, including functions related to anxiety, addiction behavior, neurotoxicity, neurodegeneration, and decision making. Finally, the role of p38 kinases in psychiatric and neurological conditions and the current progress on therapeutic inhibitors targeting p38 kinases are covered and implicate p38 kinases in a multitude of CNS-related physiological and disease states.

## Introduction

Extracellular and environmental stimuli need to be integrated for adequate cellular and organismal responses. Signal transduction is a key mechanism to translate stimuli into intracellular responses that converge in alterations in gene expression, of cellular shape and motility, and of metabolic activity (Hynes et al., 2013). Mitogen-activated protein (MAP) kinases are central to multiple signal transduction pathways across a variety of cell types, including cells of the central and peripheral nervous systems (Morrison, 2012), and are evolutionarily conserved across phyla (Widmann et al., 1999). Among the three canonical MAP kinase classes are extracellular signal-regulated kinases (ERKs), c-Jun N-terminal kinases (JNK), and p38 MAP kinases (Avruch, 2007; Morrison, 2012). This review will focus on the functions of p38 MAP kinases in cells of the nervous system.

### The p38 MAP Kinase Gene Family

**FIGURE 1:**
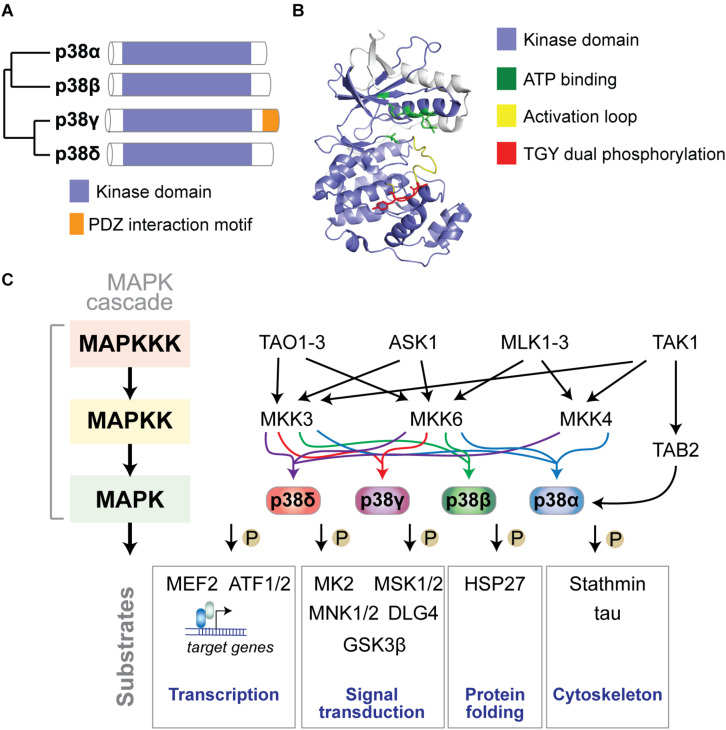
The p38 MAP kinase signaling pathway. **(A)** Dendrogram of the four mammalian p38 MAP kinases p38α, p38β, p38γ, and p38δ. Kinase domain and unique PDZ interaction motif in p38γ are highlighted. **(B)** Structure of p38α showing N- and C-lobes of the kinase domain with the active site in between. ATP binding site, activation loop and TGY dual phosphorylation are highlighted. The active site is covered by the activation loop, which undergoes dual phosphorylation resulting in conformation change, access of substrates to the active site and full kinase activation. Structure was reproduced from PDB 3HVC using Pymol. **(C)** Mammalian p38 kinases comprise of 4 proline-directed serine/threonine kinases - p38α, p38β, p38γ, and p38δ – encoded in 4 individual genes. p38 MAP kinases are activated by a plethora of extrinsic (extracellular) or intrinsic (intracellular) factors. The classic activation of p38 follows a three-tiered mechanism. Stimuli of p38 activation are cellular stressors such as oxidative stress, inflammatory stimuli/cytokines, UV radiation, and osmotic pressure at cell membranes. Upstream stimuli activate a MAP kinase kinase kinase (MAPKKK) such as ASK1, TAK1, MLK or TAO kinases, which in turn phosphorylate and activate a MAPKK, such as MKK3, MKK4 or MKK6, which in turn phosphorylates p38 kinases on Threonine and Tyrosine residues in the activation loop. Dual phosphorylation of p38 can be detected by phospho-specific antibodies as a marker of activated p38. Dual-phosphorylated p38 is fully active and targets downstream phosphorylation substrates to alter their structure, activity, function, localization or interaction with other biomolecules. An alternative activation mechanism of p38α downstream of TAK1 involves direct interaction with TAB2 followed by auto-phosphorylation of p38α. Classic downstream targets of p38 kinases include transcription factors (e.g., ATF-1/2 and MEF2), chaperones (e.g., HSP27), cytoskeletal regulators (stathmin, tau), and other signaling factors (e.g., MAPKAP kinase 2/3, MSK1/2, MNK1/2, DLG4, and GSK3β). Feedback inhibition of p38 kinases occurs through dephosphorylation by MAP kinase phosphatases (e.g., MKP1), limiting activity of upstream kinases, or transcriptional feedback.

p38 MAP kinases are encoded in four separate genes in mammalian genomes: p38α by the *MAPK14* gene, p38β (*MAPK11*), p38γ (*MAPK12*), and p38δ (*MAPK13*) (Cuadrado and Nebreda, 2010) (Figure 1A). The structure of p38 kinases consists of N- and C-lobes of the kinase domain with the active site in between ATP binding site and TGY dual phosphorylation site. The active site is covered by the activation loop, which undergoes dual phosphorylation resulting in conformation change, permitting access of substrates to the active site and full kinase activation (Figure 1B).

Mammalian p38 genes arose from a common ancestor related to the single, orthologous *HOG1* gene in yeast (Han et al., 1994), and p38-encoding genes occur in organisms of different taxa in increasing number (Table 1). The four mammalian p38 genes emerged from tandem and segmental gene duplications (Li M. et al., 2011), and genes *MAPK14–MAPK11* and *MAPK12*–*MAPK13*, are co-located on chromosomes 22 and 6, respectively (Li S. et al., 2011). Mammalian p38 proteins exhibit a high degree of similarity with 60 percent sequence identity between the four kinases (Jiang et al., 1997) and are expressed as one single gene product per gene (Kyriakis and Avruch, 2001), with the exception of p38α (Lee et al., 1994; Yagasaki et al., 2004; Casar et al., 2007).

**TABLE 1:** p38 homologs in different model organisms and their sequence identity to human p38α.

Organism	Kinase	Identity to Hs p38α (%)	Gene name
Human (*Homo sapiens*)	p38α	100	*MAPK14*
	p38β	72.8	*MAPK11*
	p38γ	60.2	*MAPK12*
	p38δ	58.5	*MAPK13*
Mouse (*Mus musculus*)	p38α	99.4	*Mapk14*
	p38β	73.3	*Mapk11*
	p38γ	59.7	*Mapk12*
	p38δ	59.7	*Mapk13*
Fruitfly (*Drosophila*	p38a	67.2	*p38a*
*melanogaster*)	p38b	68.2	*p38b*
	p38c	44.1 (hERK1/2 37.1/36)	*p38c*
Nematode	PMK-1	61.8 (hERK1/2 41.7/41.4;	*pmk-1*
(*Caenorhabditis elegans*)		hJNK1 36.9)	
	PMK-2	48.2 (hERK1/2 37.1/36)	*pmk-2*
	PMK-3	32.8 (hERK1/2 27.8/27.6)	*pmk-3*
Yeast (*Saccharomyces*	HOG1	40.4 (hERK1/2 34.5/36.7)	*hog1*
*cerevisiae*)			

### Expression of p38 MAP Kinases

Expression of p38α in different tissues is ubiquitous compared with that of p38β, p38γ, or p38δ (Cuenda and Rousseau, 2007; Cuadrado and Nebreda, 2010; Consortium, 2015; Uhlen et al., 2015). The four p38 isoforms were detected in whole mouse brain and cerebellum, while mainly p38α and p38β localize to cells in the murine neocortex and hippocampus, with lower levels of p38γ or p38δ (Kato et al., 2000; Beardmore et al., 2005). p38α and p38β are found in most cell types of the central nervous system, including in neurons, astrocytes, microglia, endothelial cells, and spinal cord (Da Silva et al., 1997; Lee S.H. et al., 2000; Nito et al., 2008; Fitzsimmons et al., 2010; Lawson et al., 2013; Haines et al., 2015). p38γ is expressed at relatively higher levels in muscle and liver, yet is found in most tissues (Cuenda and Rousseau, 2007). p38γ expression in brain is lower compared with other organs and it is found mainly in large pyramidal neurons of the cortex and hippocampus (Ittner et al., 2016) (A Ittner, *unpublished results*). Expression of p38δ is more restricted compared with other p38 kinases and is found in exocrine and endocrine pancreas, brain, lung, heart, and myeloid granulocytes (Sumara et al., 2009; Ittner et al., 2012; Gonzalez-Teran et al., 2016). Thus, all four p38 kinases are present in the CNS, however, they serve non-redundant functions depending on cell type and context.

### p38 MAP Kinase Signaling

p38 MAP kinases are engaged by a multitude of stimuli in a context- and cell type-dependent manner (Figure 1C). p38 was originally discovered as protein kinase activated in response to lipopolysaccharide (LPS) and osmotic stress (Han et al., 1994) and in regulation of cytokine biosynthesis (Lee et al., 1994). Work over the last 25 years established that p38 regulates various cellular functions such as metabolism, secretion, migration, differentiation, apoptosis, and senescence (Boulton et al., 1991; Herlaar and Brown, 1999; Wada and Penninger, 2004). p38 is activated by cellular stressors such as inflammatory cytokines (e.g., IL-1 and TNF-α), UV irradiation, osmotic pressure, and oxidative stress (Freshney et al., 1994; Han et al., 1994; Lee et al., 1994; Rouse et al., 1994; Raingeaud et al., 1995; Borisova et al., 2018). Upstream stimuli engage a three-tiered hierarchical phosphorylation cascade to activate p38, involving sequential activation of a MAP kinase kinase kinase (MAP3K), a MAP2K, and finally the p38 MAP kinase (Zarubin and Han, 2005). The p38-specific MAP2Ks MKK3 and MKK6 are dual-specificity kinases that phosphorylate Threonine (T) and Tyrosine (Y) residues within the Thr-Gly-Tyr motif of the activation loop of p38 (Zarubin and Han, 2005). Dual phosphorylation results in structural changes within the kinase domain and increased activity of p38 (Enslen et al., 1998; Alonso et al., 2000; Brancho et al., 2003). Scaffolding proteins (e.g., JIP4) recruit upstream MAP2Ks and MAP3Ks to potentiate activation of p38 kinases (Kelkar et al., 2005). Alternative modes of activation have been described, which are mediated by protein interaction, small molecules, and both tyrosine- and autophosphorylation of p38 (Ge et al., 2002; Mittelstadt et al., 2005; Salvador et al., 2005; Gills et al., 2007). Downstream targets of p38 include cytoskeletal and scaffold proteins, transcription factors, molecular chaperones, metabolic enzymes, and signaling factors (Tannenbaum et al., 1988; Goedert et al., 1997; Parker et al., 1998; Kuma et al., 2004; Sabio et al., 2005). p38 is regulated by localization, e.g., between the cytoplasm and nuclear compartment (Kotlyarov et al., 2002; Cheung et al., 2004; Gong et al., 2010) and inactivated by transcriptional feedback, termination of stimuli, inhibition of upstream kinases or direct dephosphorylation by MAP kinase dual-specificity phosphatases (DUSPs) (Jeffrey et al., 2007; Patterson et al., 2009). DUSPs specifically dephosphorylate threonine and tyrosine residues within the MAPK activation loop, including in p38, thus negatively regulating their activation (Caunt and Keyse, 2013). Several DUSPs have been found to target p38, including DUSP12, which regulates macrophage responses (Cho et al., 2017), DUSP1, DUSP8, DUSP10, and DUSP16, which are specific for p38 and JNK (Muda et al., 1996; Theodosiou and Ashworth, 2002).

## Central Methods to Study p38 Kinases

### Small Molecule Inhibitors

Development of new kinase inhibitors is critical to studying protein kinase function (Fabian et al., 2005). Small molecule inhibitors are key tools to study p38 function in many cell types including within the CNS (Table 2). The 1st generation of p38 inhibitors are based on imidazole-pyrimidine scaffolds (Scior et al., 2011) and include SB239063 (Underwood et al., 2000), SB202190 (Nemoto et al., 1998), and SB203580 (Cuenda et al., 1995), which all target p38α and p38β at concentrations used in most experimental conditions, yet show no activity toward p38γ or p38δ (Cuenda et al., 1995). Additionally, specificity limitations of 1st generation p38 inhibitors led to off-target effects in autophagy, metabolism, and glucose uptake (Borsch-Haubold et al., 1998; Bazuine et al., 2004; Fabian et al., 2005; Menon et al., 2015). This prompted development of inhibitors that target all forms of p38, such as BIRB-796 (Regan et al., 2003), or compounds that are selective toward one specific p38 kinase, such as VX-745, a selective p38α inhibitor (Alam et al., 2017). The number of isoform-specific p38 inhibitors has since increased, including VX-702 (Damjanov et al., 2009), MW-108, MW-181 (Watterson et al., 2013), SCIO 469 (Hideshima et al., 2004), and BMS 582949 targeting p38α (Emami et al., 2015) (see Table 2). Development of isoform-specific compounds was based on more targeted inhibitor design strategies (Lee and Kim, 2017; Rohm et al., 2019). Nevertheless, non-specific effects of pharmacological p38 kinase inhibitors on other kinases or off-target effects, demand more specific targeting of individual p38 kinases.

**TABLE 2:** Examples of p38 MAP kinase inhibitors used in research.

Inhibitor	p38 specificity	Chemical class	IC50	Reference	Off-target effects (including ref.)	Target disease models
SB203580	p38α/β	Imidazolidine	For p38α = 50 nM, for p38β = 500 nM,	Lee et al., 1994; Cuenda et al., 1995	GLUT1/4 (Antonescu et al., 2005), autophagy (Menon et al., 2015), CK1 (Shanware et al., 2009)	Endometriosis, inflammation, AD
MW069	p38α	Pyridazine	For p38α = 21 nM	Song et al., 2013		ALS
BIRB796	p38α/β/γ/δ	Pyrazoles	For p38α = 38 nM, for p38β = 65 nM, for p38γ = 200 nM, and for p38δ = 520 nM	Regan et al., 2003		Crohn’s disease (Schreiber et al., 2006) Psoriasis Rheumatoid arthritis Hypertension
VX-745	p38α	Pyrimidopyridazines	For p38α = 10 nM, for p38β = 220 nM	Alam et al., 2017		AD, Inflammation (Xu et al., 2010), Rheumatoid or osteoarthritis (Gibson et al., 2008), Werner syndrome
SD-282	p38α	Indolecarboxamide	For p38α = 1.61 nM	Sweitzer et al., 2004		Nociception, neuropathic pain, Diabetes, Arthritis, Myocardial injury, Sepsis, Asthma
SB220025	p38α	Imidazolidine	For p38α = 60 nM	Jackson et al., 1998		AD, Angiogenesis, Chronic Inflammation, Arthritis
MW151	p38α	Pyridazine	For p38α = 50 nM	Amantini et al., 2007		AD, Inflammation, Multiple Sclerosis
Ro3206145	p38α/β	4-Azaindoles	For p38α = 10 μM	Dapper et al., 2013		Optic neuropathy (Dapper et al., 2013)
VX-702	p38α	Pyridinecarboxamide	For p38α = 20 nM	Damjanov et al., 2009		Rheumatoid arthritis (Damjanov et al., 2009)
MW-108, MW-181	p38α	Pyridazine-3-amine	For p38α = 30 nM	Watterson et al., 2013		Neuroprotection against neurotoxic insult (Xing et al., 2015)
SCIO 469	p38α	Indole-3-acetamide-hydrochloride	For p38α = 9 nM	Hideshima et al., 2004		Monoclonal gammopathies (Roccaro et al., 2008), multiple myeloma (Hideshima et al., 2004)
BMS 582949	p38α	Triazine-6-carboxamide	For p38α = 13 nM	Emami et al., 2015		Arterial inflammation (Emami et al., 2015)

### Gene Targeting Approaches

Genetic modification of individual *p38* genes has been instrumental in uncovering non-redundant functions of individual p38 kinases (Table 3). Two main strategies have been employed: (1) conventional gene targeting in mice to generate complete or conditional knockout alleles for *p38*α (Hui et al., 2007; Ventura et al., 2007), *p38*β (Beardmore et al., 2005; Xing et al., 2015), *p38*γ (Pogozelski et al., 2009), *p38*δ (Sumara et al., 2009; Risco et al., 2012). Global deletion of *p38* genes in mice results in viable offspring, with the exception of complete *p38*α knockout, which results in embryonic lethality due to placentation defects (Adams et al., 2000). (2) Gene editing approaches using the CRISPR-Cas system to target p38 genes in cells or mice (Goginashvili et al., 2015; Stefanoska et al., 2018).

**TABLE 3:** Mouse models of *p38* gene targeting in and affecting functions of CNS cells.

p38 gene	Mouse strain	Cell type targeted	Phenotype(s)	Potential disease relevance	References
*p38α (Mapk14)*	*Thy-cre p38*α^loxP/loxP^	CNS neurons	Increased anxiety-related behavior	Depression, anxiety	Stefanoska et al., 2018
	*CamK2a-cre p38*α^loxP/loxP^	Neurons	Reduced Aβ accumulation, Reduced tau pathology	Tauopathies, Alzheimer’s disease	Schnoder et al., 2016; Colie et al., 2017
	*ePet1-cre p38*α^loxP/loxP^	Serotonergic neurons, Raphe nucleus neurons	Increased stress resilience, altered 5HT reuptake	Depression, anxiety, addiction, schizophrenia and autism	Bruchas et al., 2011; Lemos et al., 2012; Schindler et al., 2012
	*Nes-creERT2 p38*α^loxP/loxP^	Neural progenitor cells	Reduced neural progenitor proliferation	Age-related neurodegeneration	Kase et al., 2019
	*Gfap2-cre p38*α^loxP/loxP^	Astrocytes	Reduced cytokine release from astrocytes, altered serotonin uptake	Neuroinflammation	Bruchas et al., 2011; Lo et al., 2014
	*Lys-cre p38*α^loxP/loxP^; *Cx3cr1-cre p38*α^loxP/loxP^;	Microglia	Reduced cytokine release from microglia	Neuroinflammation, traumatic brain injury, Alzheimer’s disease	Bachstetter et al., 2011, 2013; Morganti et al., 2019
	*Ng2-cre p38*α^loxP/loxP^; *mP_0_TOTA*-cre *p38*α^loxP/loxP^	Myelinating cells	Inhibition of myelination during development and remyelination	Nerve injury, multiple sclerosis	Chung et al., 2015, 2018; Roberts et al., 2017; Chung et al., 2018
*p38*β (*Mapk11*)	*p38β^–/–^*	Global	No CNS phenotypes reported		Xing et al., 2015
*p38*γ (*Mapk12*)	*p38γ^–/–^*	Global	Increased susceptibility to excitotoxicity	Alzheimer’s disease	Ittner et al., 2016
	*p38γ^–/–^*	Global	decreased peripheral nerve myelin caliber	Multiple sclerosis	Noseda et al., 2016
*p38*δ (*Mapk13*)	*p38δ^–/–^*	Global	No CNS phenotypes reported		Sumara et al., 2009; Ittner et al., 2012

### RNA Interference

Expression of siRNA or shRNA targeting p38 transcripts offers a feasible solution for reducing p38 levels when neither kinase inhibitors nor genetic alteration on the DNA levels is possible or desired. RNA interference has been used for all *p38* transcripts in the past (Sumara et al., 2009; Yang et al., 2013; Stefanoska et al., 2018) and offers gene-specific reduction in p38 transcript and protein levels. Cell type-specific or brain region-specific isoform-selective RNA interference likely will see increased use to explore functions of p38-regulated pathways *in vivo* in future studies.

### Protein Variants of p38

Constitutively active (CA) variants of all human p38 kinases have been engineered by targeted codon exchanges in the coding sequences (Avitzour et al., 2007). For example, exchange of the Asp-179 to Alanine residue in the activation loop in p38γ results in increased kinase activity, likely due to enhanced engagement with upstream activating kinases (Avitzour et al., 2007). In addition to codon exchanges near the activation loop, CA variants for p38α and p38δ have been generated by single residue exchange at Tyr-323 (Diskin et al., 2004), a region that is associated with tyrosine kinase-mediated p38α activation by ZAP-70 (Salvador et al., 2005). CA variants have been used in cell culture to study effects of downstream target phosphorylation or of increased kinase activity itself (Sumara et al., 2009; Ittner et al., 2016). p38^CA^ expression by viral vectors or through transgenesis can be employed to address functions of p38 kinases and their activity in primary cells, organ culture or *in vivo* (Ittner et al., 2016). Dominant negative (DN) variants of p38 can be generated by mutations in the activation loop changing the dual phosphorylation motif into Ala-Gly-Phe, which cannot be phosphorylated and activated by upstream kinases. This approach has been used to study function of p38α in mice using a transgenic or knock-in approach (Ren et al., 2005; Cortez et al., 2017).

New biochemical methods to label protein interactions and substrate engagement facilitate mapping of entire MAP kinase signaling cascades (Prikas et al., 2020). In summary, the molecular and genetic toolbox outlined above, including inhibitor molecules, gene knockout, CRISPR, RNAi, constitutively active (CA) p38 variants, and proximity labeling, facilitated the discovery of functions of p38 kinases and will be essential for future advances toward a better understanding of p38 kinases in CNS cell types and diseases.

## Neuronal Functions of p38 Kinases

**FIGURE 2:**
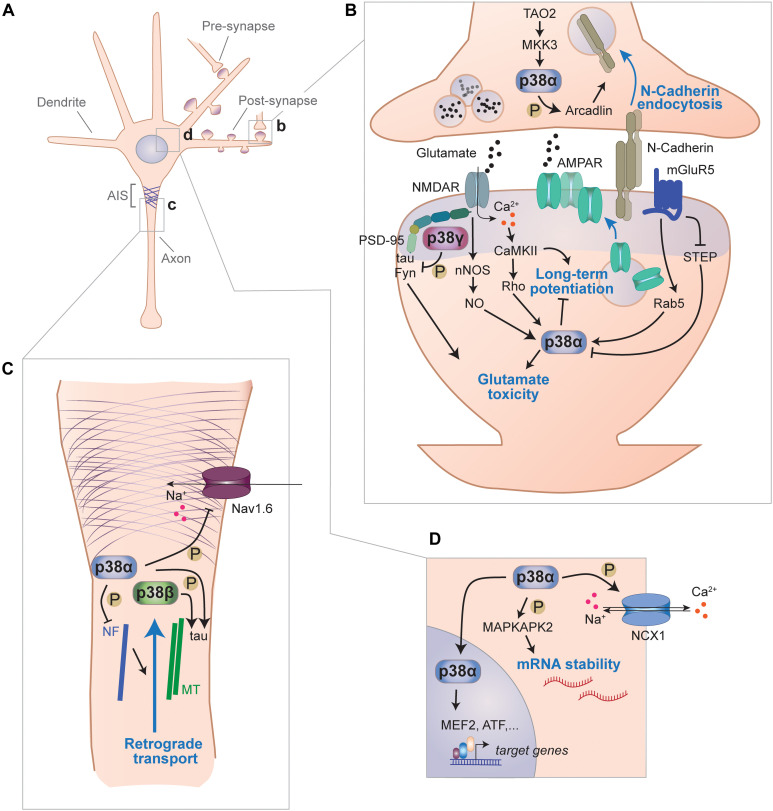
Signaling functions of p38 kinases in neurons. **(A)** Functions of p38 kinases in neurons have been described for different subcellular locales, including pre- and postsynapse, axon and cell body. **(B)** p38 kinases are involved in signal transduction at synapses. Within the pre-synaptic moiety of synapses, p38α phosphorylates arcadlin to promote N-Cadherin endocytosis from the active zone when activated downstream of TAO2 and MKK3. p38α is engaged downstream of multiple post-synaptic pathways and impairs LTP, which involves increasing surface expression of AMPA-type glutamate receptors (AMPAR) and promotes toxicity from excess glutamate. In this context, p38α is activated downstream of NMDA receptors through neuronal nitric oxide synthase (nNOS)-mediate nitric oxide (NO) production or through CaMKII) activity. Alternatively, p38α is activated downstream of metabotropic glutamate receptor 5 (mGluR5) through small G-protein Rab5 and/or inhibition of striatal enriched phosphatase (STEP). p38γ, which binds to PSD-95 within the post-synaptic density, phosphorylates tau and inhibits glutamate excitotoxicity mediated by tau and Fyn kinase. **(C)** p38 kinases are involved in axonal signaling. p38α phosphorylate voltage-gated sodium Nav1.6 channels, which localize to the axon initial segment, and reduce their sodium currents. p38α phosphorylates neurofilaments and thereby impair retrograde axonal transport processes. Both, p38α and p38β were shown to phosphorylate the cytoskeletal protein tau that associates with axonal microtubules (MT). **(D)** p38 kinases regulate neuronal processes with the soma and nucleus. p38α regulates sodium/calcium exchange channel NCX1 by phosphorylation. p38α targets MAP kinase-activated protein kinase-2 (MK2) that promotes stability of AUC-rich element containing neuronal messenger RNAs. Furthermore, p38α regulates gene expression through nuclear targets such as MEF2 or ATF transcription factors.

Of the four p38 kinases, p38α has most extensively been studied in the context of the central nervous system (Figure 2). Neuron-specific functions of p38α are mediated through a multitude of neuronal molecules, some of them central to the roles of this cell type such as ion channels, neurotransmitter receptors, cytoskeletal and adhesion molecules. p38α is expressed in CNS neurons (Stefanoska et al., 2018), where it localizes to soma, neurites and synapses (Ittner et al., 2016). p38α is found at lower levels than other p38 kinases in neurons (i.e., p38β and p38γ (Lawson et al., 2013; Ittner et al., 2016). Similar to its function in other cell types, p38α regulates the translation machinery in neurons through downstream regulation of MAP kinase-activated protein (MAPKAP) kinases and mRNA stability (Farooq et al., 2009; Lawson et al., 2013). MAPKAP kinases, including MAPKAPK2, phosphorylate Tristetraproline to regulate mRNA stability through adenine/uridine-rich elements (Clement et al., 2011). However, the identity and extent of target mRNAs downstream of p38α and MAPKAP kinases in neurons remains to be determined.

### Long-Term Potentiation and Depression

Central mechanisms of synaptic plasticity are long-term potentiation (LTP) or long-term depression (LTD), i.e., the activity-dependent up- or downregulation, respectively, of AMPA-type glutamate receptors (AMPARs) at the post-synapse. This receptor reorganization is followed by increased or reduced excitatory currents and consequently an enhancement or reduction in synaptic strength (Huganir and Nicoll, 2013; Henley and Wilkinson, 2016). p38 is involved in regulation of glutamate neurotransmission at post-synaptic neurons (Figure 2B). Activity of p38α is modulated downstream of NMDA-type glutamate receptors (NMDARs) (Krapivinsky et al., 2004), and activation of p38α inversely correlates with the number of AMPA receptors (AMPARs) at synapses upon engagement of NMDARs (Krapivinsky et al., 2004). These findings implicate p38α in regulation of post-synaptic NMDAR signaling and AMPAR trafficking. Regulation of synaptic plasticity appears to be an evolutionary conserved function of p38. In the nematode *Caenorhabditis elegans*, trafficking of glutamate receptor GLR-1 is altered in *pmk-1* (p38 MAPK) and *sek-1* (MAPKK) mutants, which display greater GLR-1 internalization in neurons (Park and Rongo, 2016). In addition, increasing age of nematodes lowers p38 levels and reduces GLR-1 in membranes, which can be restored by increasing p38 levels (Park and Rongo, 2016). This is consistent with work in other model organisms suggesting that p38 promotes AMPAR downregulation and short- and long-term depression (Guan et al., 2003).

p38α inhibits NMDAR-mediated LTP. Experimental stimulation of NMDARs results in inactivation of p38 and relieves inhibition of NMDAR-mediated synaptic AMPAR expression (Krapivinsky et al., 2004). NMDARs engage – at least in part – a pathway through calcium-activated/calmodulin-dependent protein kinase II (CamKII) that activates and SynGAP-MUPP1 that inhibits p38α, respectively (Krapivinsky et al., 2004). This inhibition of p38α activity is concomitant with dephosphorylation of SynGAP, a negative regulator of Ras-Raf-MEK-ERK signaling (Krapivinsky et al., 2004). Interestingly, *SynGAP* knockout neurons show reduced AMPAR surface expression and yet lower p38 activation suggesting that p38 activity is either required for AMPAR surface presentation or other SynGAP-modulated pathways (deregulated in *SynGAP* knockout cells) have a dominant effect over lower p38 activity (Rumbaugh et al., 2006). Inhibition of LTP formation is mediated through p38α in different models when LTP is inhibited by angiotensin-II (Dai et al., 2016), oligomerized amyloid-beta peptides (Li S. et al., 2011; Chen et al., 2013), or interleukin-1beta (Tong et al., 2012). Genetic ablation confirmed a specific involvement of p38α in inhibition of LTP formation by amyloid-β peptides (Colie et al., 2017). Thus, evidence from multiple experimental strategies corroborate p38α as a negative regulator of LTP formation.

p38α has been implicated in the induction of LTD in different model systems. p38α enhances LTD in the rat hippocampus/dentate gyrus (Coogan et al., 1999; Rush et al., 2002; Murray and O’Connor, 2003; Stein et al., 2020). In this context, p38α is activated downstream of group I metabolic glutamate receptors (mGluR1/5) through a mechanism dependent on small GTPase Rap1, leading to Rab5-mediated AMPAR internalization and LTD formation in hippocampal neurons (Rush et al., 2002; Huang et al., 2004; Hsieh et al., 2006). A key downstream target of p38α in LTD is Rab5, a GTPase critical for the endocytic cycle of AMPARs (Brown et al., 2005). The protein tyrosine phosphatase STEP is inhibited downstream of mGluR1/5 (Moult et al., 2008). STEP can directly dephosphorylate and hence deactivate p38α (Pulido et al., 1998; Munoz et al., 2003). STEP deactivation can also be mediated by extrasynaptic NMDA receptors through calcium entry (Xu et al., 2009), which confers inhibition of LTP and induction of LTD (Hardingham and Bading, 2003). STEP is itself of therapeutic interest. Regulation of STEP is involved in LTP deficits in a mouse model of fragile X syndrome (Goebel-Goody et al., 2012) and mouse models of AD (Xu et al., 2014). The effects of modulated STEP activity though may be mediated through a multitude of pathways other than p38α (Deb et al., 2013; Xu et al., 2015). STEP is required for calcium homeostasis due to increased calcium levels and CamKII activation in hippocampal neurons from *STEP*-deficient mice (Bosco et al., 2018), suggesting STEP is an inhibitor of calcium-mediated signaling and neurotransmitter release.

Whether ionotropic properties of NMDA receptors or calcium-independent downstream signals are important for p38-mediated LTD is, however, unclear. Experiments with alterations in extracellular calcium suggest ionotropic NMDAR function is critical to activate p38α downstream of NMDA receptors (Kawasaki et al., 1997; Ahn et al., 2000; Li et al., 2006; Babiec et al., 2014). However, LTD and dendritic spine shrinkage within a short timeframe could be induced through p38 activation downstream of NMDA receptors despite a complete blockage of NMDA receptor channel properties (Stein et al., 2015, 2020), suggesting that there are additional mechanisms to activate p38 during LTD enhancement that are independent of NMDAR-mediated calcium currents (Nabavi et al., 2013, 2014). The calcium-dependent activation of p38 through NMDARs requires neuron-specific factors such as PSD-95 and neuronal nitric oxide synthase (nNOS) (Soriano et al., 2008). The interaction of nNOS with the NMDAR promotes p38 activation and neuronal excitotoxicity, a mode of toxicity driven by excessive glutamate release/stimulation (Cao et al., 2005).

Synaptic plasticity is considered a neuronal proxy of memory consolidation or extinction (Bailey et al., 2015). Although experiments using cultured brain tissue slices or isolated primary neurons supported a critical role of p38α in LTP inhibition and LTD induction, mice with neuron-specific deletion of *p38*α did not show altered or impaired memory function (Stefanoska et al., 2018).

### Regulation of Ion Channels

p38α has been implicated in modulating neuronal excitability through direct action on sodium channels (Figures 2C,D). p38α phosphorylates Ser-553 in L1 region of voltage-gated sodium channel Nav1.6 (Wittmack et al., 2005). This phosphorylation event reduces sodium current amplitude through Nav1.6 channels (Wittmack et al., 2005). p38α regulates channel Nav1.8 modulation by phosphorylation, which increases current density in dorsal root ganglion neurons (Hudmon et al., 2008). Voltage-gated sodium channels Nav1.6 and Nav1.8 are widely expressed (Goldin, 1999). Therefore, p38α-mediated sodium current modulation may be applicable to a variety of neurons involved in different brain regions and cognitive functions. In fact, p38α colocalizes with Nav1.6 in cerebellar Purkinje cells and is found in a complex with Nav channels in lysates from rat brain (Wittmack et al., 2005). This association of Nav channels with p38α may serve as a fast-acting regulatory mechanism when Nav channel currents need to be moderated. Consistent with this concept, Nav channel blocker riluzole increases p38 activation in hippocampal neurons (Kato et al., 2000; Ittner et al., 2014). Whether genetic ablation of neuronal *p38*α sufficiently disrupts regulation of Nav channels, resulting in lower voltage-gated currents and further downstream effects (e.g., on action potentials and neuronal network synchronicity) remains unclear. Multiple kinases have been reported in regulation of Nav channels (Stamboulian et al., 2010). Hence, redundancy among kinases in their action on Nav channels could mask phenotypes of individual genetic deletion of *p38*α.

p38α regulates excitability and ion currents in neurons through other channels. Based on results from neuronal PC12 cell line, NCX1-3 sodium/calcium exchange channels, which are required for maintenance of baseline calcium levels in neurons after excitation is under partial transcriptional control of p38 pathway (Sirabella et al., 2012).

In primary sensory neurons, the transient receptor potential cation channel subfamily V member 1 (TRPV1) channel levels in the plasma membrane are increased by NGF in a p38-dependent manner (Ji et al., 2002). This function of p38α may be present in other CNS neurons as TRPV1 is found in the hippocampus, where they impact synaptic plasticity and memory function (Gibson et al., 2008). TRPV1 channels were also reported to activate p38 once engaged by noxious stimuli (e.g., capsaicin) (Amantini et al., 2007). This may be a positive feedback loop that enhances TRPV1 levels and thus augments noxious signal transmission and nociception.

### Synaptic Development

Evidence from nematode studies suggests that p38 signaling may have a function in development of the presynapse. Presynaptic development requires suppression of a pathway consecutively involving DLK1, MKK4 and p38 (*pmk-3*) downstream of the RING ligase *rpm1* (Nakata et al., 2005). This may be accompanied by changes in synaptic mRNA stability and translation downstream of the DLK1-p38-MAPKAP kinase pathway (Yan et al., 2009). Interestingly, p38 targets Rab5 in the context of ciliogenesis in the nematode (van der Vaart et al., 2015). Rab5 mediates effects of p38α in LTD formation in mammalian cells (Rush et al., 2002; Huang et al., 2004; Hsieh et al., 2006). Thus, this conserved function in nematode dendritic ciliogenesis may relate to mammalian synaptic plasticity. However, the identity of the MAP kinases activated downstream of DLK may differ between invertebrate and mammalian neurons. In mammalian neurons, the DLK pathway has been linked to post-synaptic neurotoxic, axonal injury stress signaling, where DLK engages JNK downstream rather than p38 (Pozniak et al., 2013), and to neuronal migration (Hirai et al., 2006).

### Synaptic Adhesion

p38 kinases are involved in synaptic adhesion molecule expression in neurons (Figure 2B). N-cadherin – a key neuronal homotypic cell–cell adhesion molecule regulating hippocampal spine density (Benson and Tanaka, 1998) and vesicle turnover at neuronal synapses (van Stegen et al., 2017) – is downregulated upon induced neuronal activity through a pathway that involves activation of p38 downstream of TAO2 kinase (Yasuda et al., 2007). Here, p38α is likely the key p38 MAPK involved, based on the use of isoform-specific inhibition in these experiments (Yasuda et al., 2007). Downstream factors of p38 in downregulation of N-cadherin are unknown, yet may involve Rab5 similar to p38-mediated AMPA receptor endocytosis (Rush et al., 2002; Huang et al., 2004; Hsieh et al., 2006). NMDAR activity induces N-cadherin downregulation through β-catenin (Tai et al., 2007). This pathway is (at least partially) reminiscent of the TAO2 kinase-p38α pathway function that was shown to regulate N-cadherin endocytosis upon neuronal activation (Yasuda et al., 2007). Whether p38α regulates spine morphology through N-cadherin surface levels in the hippocampus of living mice remains to be determined.

### Neuronal Cytoskeleton

Regulation of cytoskeletal proteins and processes through p38 has been investigated in various cell types. Neuronal targets of p38 kinases associated with the cytoskeleton include proteins of the intermediate filaments, microtubule and actin network (Figure 2C). p38α phosphorylates neurofilament (NF) in neuronal axons (Ackerley et al., 2004). Though the physiologic function of this NF phosphorylation is unknown, phosphorylated NF colocalizes with active p38α in neurons from SOD1 mouse model of ALS and in ALS patient samples. p38 kinases can affect the neuronal microtubule network through several targets. A key target is the microtubule-associated protein tau. p38α, p38β, p38γ, and p38δ phosphorylate recombinant tau simultaneously on multiple sites *in vitro* (Goedert et al., 1997; Cavallini et al., 2013). *In vitro* experiments suggest that the phosphorylation of tau by p38 alters microtubule assembly (Feijoo et al., 2005). p38 kinases may phosphorylate markedly different sites on substrate proteins in cells *in vivo* than on recombinant protein isolated in the lab. Neuronal expression of constitutively active p38γ results in site-specific phosphorylation at a few sites on tau (Ittner et al., 2016). p38 kinases may regulate the microtubular cytoskeleton through different pathways than tau. The tubulin glutamate ligase TTLL-4 was shown to be activated by p38 (pmk-1) in ciliated sensory neurons of the nematode *C. elegans* (Kimura et al., 2018). Whether p38 kinases regulate microtubule assembly and tubulin glutamylation in mammalian neurons *in vivo* remains to be determined.

Regulation of the actin cytoskeleton by p38 kinases underlies the functions of p38 identified in neuronal migration. Neuronal migration of gonadotropin-releasing hormone-expressing neurons is mediated through p38α and downstream MAPKAP kinase-Hsp25-mediated regulation of actin dynamics (Allen et al., 2002). Despite this function identified in a specific neuronal precursor type, p38s have been implicated more broadly in a signaling network that controls cortical neuron migration (Segarra et al., 2006). Whether this involvement depends on developmental stage and cortical layer is unclear as is the relevance to gross cortical development or the identity of the p38 kinase(s) involved. Notably, deletion of *p38*α in forebrain neurons late in development and postnatally does not result in compromised cortical layer microanatomy (Stefanoska et al., 2018). Additional systematic studies will be required to address the role of p38 kinases in migration of neuronal precursors or specialized neurons within the CNS of model organisms at different stages of their development.

### Axonal Maintenance and Transport

p38 kinases may serve localized roles in the axon of neurons (Figure 2C). Both p38α and p38β have at least partial axonal localization in cultured neurons (Ittner et al., 2016). Axonal injury experiments in cultured neurons support a function of JNK and p38 activation during axon regeneration (Nix et al., 2011). Activation of both MAP kinase classes was regulated at different stages of axonal regeneration. This sequential engagement of JNK and p38 kinase may reflect to some extent the invertebrate JIP1/DLK pathway involved in axonal injury and in physiological functions at synapses (Klinedinst et al., 2013). This indicates mammalian diversification of MAP kinase signaling in the axon.

Increasing evidence suggests that p38 kinases are involved in regulation of axonal transport. Kinesin function is inhibited by p38-dependent activation of kinesin light chain kinases (De Vos et al., 2000). This role of p38 activity in the axon was confirmed in a squid neuron model, a classic experimental system to study axonal transport (Song et al., 2013). Activation of p38 downstream of ASK1 interfered with axonal transport of mutant SOD1 protein, likely worsening symptoms of ALS (Morfini et al., 2013; Song et al., 2013). These results were recently supported by inhibitor experiments in a mouse model of ALS, suggesting that the p38 kinases involved in axonal transport inhibition is p38α (Gibbs et al., 2018). The p38α inhibitor MW069 restored axonal transport and outgrowth in FUS-expressing motor neurons (Sama et al., 2017), supporting again that p38α has an important role in regulation of axonal transport processes, at least under disease conditions. This function of p38α may also contribute to axonal impairments in optic neuropathy (Dapper et al., 2013). Insights into the role of p38 kinases, and in particular p38α, in axonal transport from inhibitor studies should be further corroborated by future experiments with genetic ablation.

### Neurotoxicity and Survival

A bulk of studies based on p38 inhibitor experiments support a central function of p38 kinases in neurotoxic signaling or neurotoxicity in general. Most of this work explored the role of p38 kinases in cultured neurons. Specific inhibition of p38α is neuroprotective in multiple forms of neurotoxicity, while p38β does not mediate neurotoxic signals (Xing et al., 2015). This function of p38α may apply across different neuronal cell types. p38α inhibition, for example, protected motoneurons from hypoxia-induced cell death (Guo and Bhat, 2007) or 1-methyl-4-phenyl-1,2,3,6-tetrahydropyridine (MPTP)-induced dopaminergic neuron loss (Wu et al., 2016). Excitotoxicity, a complex form of toxicity initiated by excess glutamate levels (Hardingham and Bading, 2003), is at least in part mediated through calcium- and Rho GTPase-mediated activation of p38α, resulting in excitotoxic cell death of cultured neurons (Semenova et al., 2007). Prions (Prp^Sc^) and prion-like amyloid-beta oligomers mediate some of their neurotoxic effect in cultured hippocampal neurons through a mechanism that involves NMDA receptor, calcium and p38 activation (Fang et al., 2018). This is partially reminiscent of excitotoxic signaling and these modes of neurotoxicity show substantial overlap in signaling molecules involved, e.g., downstream of NMDA receptors (Hardingham and Bading, 2003). Hence, many pathways that mediate NMDA receptor-mediated synaptic plasticity are also involved in glutamate excitotoxicity (Hardingham and Bading, 2003; Mucke and Selkoe, 2012). Upstream factors of p38 in synaptic plasticity, such as nNOS, calcium, STEP, Rho, and Rab5 affect the role of p38 in glutamate toxicity (Figure 2B). Other experimental methods used to induce glutamate-dependent excitotoxicity have included blocking inhibitory neuron function [e.g., by blocking GABA receptors (Cremer et al., 2009) or engaging kainic acid (KA) receptors (Chittajallu et al., 1996)] with KA to induce seizures (Namiki et al., 2007). Our systematic study of different p38 knockout mice, including neuron-specific *p38*α knockout mice, using PTZ-induced glutamate toxicity as model, did not find involvement of p38α, however, found p38γ is important in suppression of glutamate toxicity (Ittner et al., 2016).

NMDA receptor-mediated glutamate toxicity involves tau-dependent recruitment of Fyn kinase into a complex of NMDA receptor with scaffolding factor PSD-95 (Ittner and Ittner, 2018). PSD-95 serves as a postsynaptic hub for protective and toxic signaling cascades (Cao et al., 2005; Ittner et al., 2010, 2016). Fyn kinase phosphorylates NMDAR subunit NR2B on Tyrosine-1472, which aggravates excitotoxicity (Aarts et al., 2002). This pathway is inhibited by p38γ, which localizes to post-synapses and binds to PSD-95 through a PDZ domain interaction (Sabio et al., 2005; Ittner et al., 2016). p38γ phosphorylates dendritic tau on Threonine-205, which abolishes a complex of PSD-95, tau and Fyn kinase (Ittner et al., 2016). Therefore, p38γ has a neuroprotective function in excitotoxicity and in other related forms of neurotoxicity such as amyloid-β exposure (Ittner and Ittner, 2018). Whether p38γ protects neurons from other noxious stimuli and whether these functions of p38γ are all mediated through tau phosphorylation remains to be determined. Thus, depending on context – p38 kinases can have roles that promote survival of neurons and are likely linked to physiologic mechanisms e.g., for p38α or p38γ in NMDA receptor signaling. p38α was shown to promote neuronal survival in culture as a downstream factor of Rit GTPase (Cai et al., 2012) and proliferation of cerebellar granule neuron precursor (Guldal et al., 2012). Overall however, neuron-specific *p38*α or global deletion of *p38*β, *p38*γ, *p38*δ did not result in different neuronal numbers under physiological conditions (Ittner et al., 2016; Colie et al., 2017; Stefanoska et al., 2018). Effects of p38 kinases in neurotoxicity or -protection become apparent upon exposure to noxious stimuli. Thus, studies in cultured neurons should be systematically translated to KO models to confirm relevance of the findings. Consistently, a rigorous approach supports a central function of JNK in neuronal apoptosis following excitotoxic insult rather than p38α (Cao et al., 2004). Deletion of neuronal *p38*α or global deletion of *p38*β, *p38*δ did not result in altered susceptibility to excitotoxicity in mice (Ittner et al., 2016). Deletion of *p38*γ aggravates glutamate toxicity, and neuron-specific transgenesis of CA *p38*γ protects mice and cells from excitotoxicity, reflecting the protective function of p38γ (Ittner et al., 2016). Notably, neuron-specific CA *p38*γ transgenic mice show no obvious cognitive or behavior phenotypes (Ittner et al., 2016). Thus, depending on context, neuronal p38 kinases serve signaling functions that contribute to resolution of stress through their activity.

### Functions in Neuronal Subtypes

p38 kinases have specific functions in neuronal subtypes. Though the functions of individual p38 kinases have not been studied across many different neuron types, there are outstanding examples in specific neuron classes.

Mice that express a dominant negative (DN) variant of p38α in neuronal progenitors show slower age-dependent decline in neurogenesis and context-dependent fear conditioning (Cortez et al., 2017). p38α was shown to regulate proliferation in peripheral cell types, including hepatocyte and hematopoietic precursor cells (Hui et al., 2007). Effects on proliferation are mediated through JNK, MAP kinase-activated protein kinase-2 (MK2), and p53 (Hui et al., 2007; Gupta and Nebreda, 2015), yet neuronal precursor mechanisms have not been explored.

A study employing targeted deletion of *p38*α in serotonergic neurons elegantly showed that p38α is required for stress resilience, a protective mechanism in depression and addiction. p38α controls serotonin (5HT) levels in 5HT neurons in the dorsal raphe nucleus (DRN) by phosphorylation and trafficking of the serotonin transporter (SERT) (Bruchas et al., 2011). Whether other p38 kinases expressed in 5HT neurons can phosphorylate SERT has not been addressed. This action of p38α results in differential 5HT re-uptake and modulation of serotonin involved in behavior related to anxiety or depression (Bruchas et al., 2011; Baganz et al., 2015). This mechanism is present in 5HT neurons across different brain regions. In the ventral striatum, SERT serotonin uptake is modulated upon stress exposure, which was shown to activate p38α downstream of κ-opioid receptors resulting in increased SERT cell surface expression (Schindler et al., 2012). 5HT cells in the dorsal raphe nucleus respond to exposure of rodents to stress with activation of κ-opioid receptors and p38α (Lemos et al., 2012). Consistently, 5HT neuron specific deletion of p38α or administration of p38α inhibitor (MW150) restores phenotypes of a SERT mutant mouse model of social defeat stress (Robson et al., 2018), and pan-neuronal deletion of p38α resulted in reduced resilience to novelty stress and an altered anxiety response (Stefanoska et al., 2018). Pan-neuronal p38α-deficient mice had increased activity of JNK in the forebrain and stress response in these mice was restored by inhibition of JNK (Stefanoska et al., 2018). These pan-neuronal effects are likely to occur in combination with effects of p38α activity in serotonergic neurons to achieve tailored responses to aversive stimuli and stress.

Similarly, p38α acts in dopaminergic neurons to regulate aspects of behavioral inhibition. κ-opioid receptor engage p38 activation in DA neurons of the ventral tegmental area (VTA) (Abraham et al., 2018) and this pathway is critical in VTA DA neurons for regulating aversive behavior (Ehrich et al., 2015). Given the similarities between DA and 5HT transporters, these effects are potentially regulated through targeting of DA transporter trafficking and function by p38 (Foster et al., 2006).

In addition, p38 controls DA neuron survival and resistance to oxidative stress. p38 activity downstream of ASK1 is inhibited by klotho (Brobey et al., 2015b), a factor that prevents premature aging and striatal DA neuron loss (Hsieh et al., 2010; Kosakai et al., 2011) and promotes remyelination (Kosakai et al., 2011; Zeldich et al., 2015). Thus, inhibition of p38 by klotho may underlie protection from striatal DA neuron loss (Kosakai et al., 2011). Activation of ASK1 is controlled by calcium through binding and inhibition by the calcium-binding protein CIB1 through direct interaction (Yoon et al., 2017). ASK1 is a key upstream activator of p38 kinases in oxidative stress signaling in multiple cell types (Watanabe et al., 2015). Therefore, toxicity mechanisms that engage the ASK1-p38 pathway in DA neurons may depend on generation of reactive oxygen intermediates. Specific functions of other p38 kinases present in DA neurons are unknown, yet at minimum p38β can be activated downstream of ASK1 (Mizumura et al., 2006). Systematic investigation of expression and function of p38β and p38γ in DA neurons may contribute to our understanding of p38-dependent mechanisms in this neuron type.

p38 kinases were shown to be involved in basic mechanisms within clock neurons that regulate diurnal rhythms in organisms across phyla. Fruit flies deficient in *p38b* or overexpressing p38b in clock neurons showed opposite arrhythmic circadian behavior. p38b controls period gene expression programs in clock neurons through downstream transcription factor Mef2 – an established nuclear target of p38 (Zhao et al., 1999) – and kinase MNK (Dusik et al., 2014; Vrailas-Mortimer et al., 2014). In vertebrates, active p38 was detected in pineal gland neurons, suggesting p38 kinases are involved in circadian periodicity (Hayashi et al., 2003). It is unclear how p38 kinases are involved in neurons in controlling the circadian rhythm in the mammalian brain. Studies in *p38* knockout mice may inform whether lack of p38 kinases affects gross circadian rhythms in terms of activity, sleep, and metabolism.

### Neuronal Differentiation

Apart from functions in specific neuron types, p38 kinases contribute to the control of neuronal differentiation and transdifferentiation. Transdifferentiation of sympathetic neurons into cholinergic neurons was shown to be regulated by p38α and p38β *in vitro* (Loy et al., 2011). Experiments with neurospheres derived from adult hippocampal neural stem cells suggest that p38α controls neuronal differentiation and inhibition of p38α maintains pluripotency of progenitor cells (Yoshioka et al., 2015). However, p38 kinases appear dispensable for initial differentiation into neuronal cell types *in vivo*. Neuronal knockout of *p38*α and global knockout of *p38*β or *p38*γ does not result in gross reduction of neuronal numbers (Xing et al., 2013; Ittner et al., 2016; Maphis et al., 2016; Schnoder et al., 2016; Colie et al., 2017). These results may, however, not be conclusive to p38α function for early neuronal differentiation processes in embryogenesis due to timing and restriction of cre recombinase expression (Dewachter et al., 2002). However, experiments in cultured cells have produced conflicting results. *p38*α-deficient multipotent embryonic stem cells can undergo induced differentiation into neurons (Guo et al., 2007), suggesting p38α is dispensable for neuronal differentiation, potentially based on compensation by other p38 kinases. Other results support that differentiation from murine embryonic stem cells is partially dependent on p38α and transcriptional control of *bcl2* (Aouadi et al., 2006; Trouillas et al., 2008). NGF promotes neuronal differentiation through Rin GTPase and p38α (Shi et al., 2005). Inhibition of p38α may indirectly contribute to efficient differentiation through supporting survival of differentiating neurons (Okamoto et al., 2000; Wu et al., 2010). Future studies should explore whether there are indeed non-redundant pathways dependent on p38 kinases in neuronal differentiation processes that are not solely related to stress signaling or confounding factors in cultured system. More systematic single neuron analysis with early or global ablation of p38 genes may give a broad picture of roles of these kinases in differentiation in the developing brain.

## Astrocytic Functions of p38 Kinases

While much work has focussed on neurons, the p38 family of MAP kinase has important functions in astrocytes. Astrocytes contribute to synaptic transmission by clearance of neurotransmitters, neurotransmitter metabolism and provide structural components of the tripartite synapse architecture, which is made up of a pre-synaptic, a post-synaptic neuronal as well as an astrocytic component in the mammalian central nervous system (Halassa et al., 2007; Santello et al., 2012). Thus, functions of signaling pathways in astrocytes can be relevant to cognition, memory, behavior, development and disease states of the CNS. At least p38α and p38β are present in astrocytes (Piao et al., 2002; Lo et al., 2014). However, systematic expression studies of all four p38 kinases in astrocytes during resting and stimulated conditions are missing.

**FIGURE 3:**
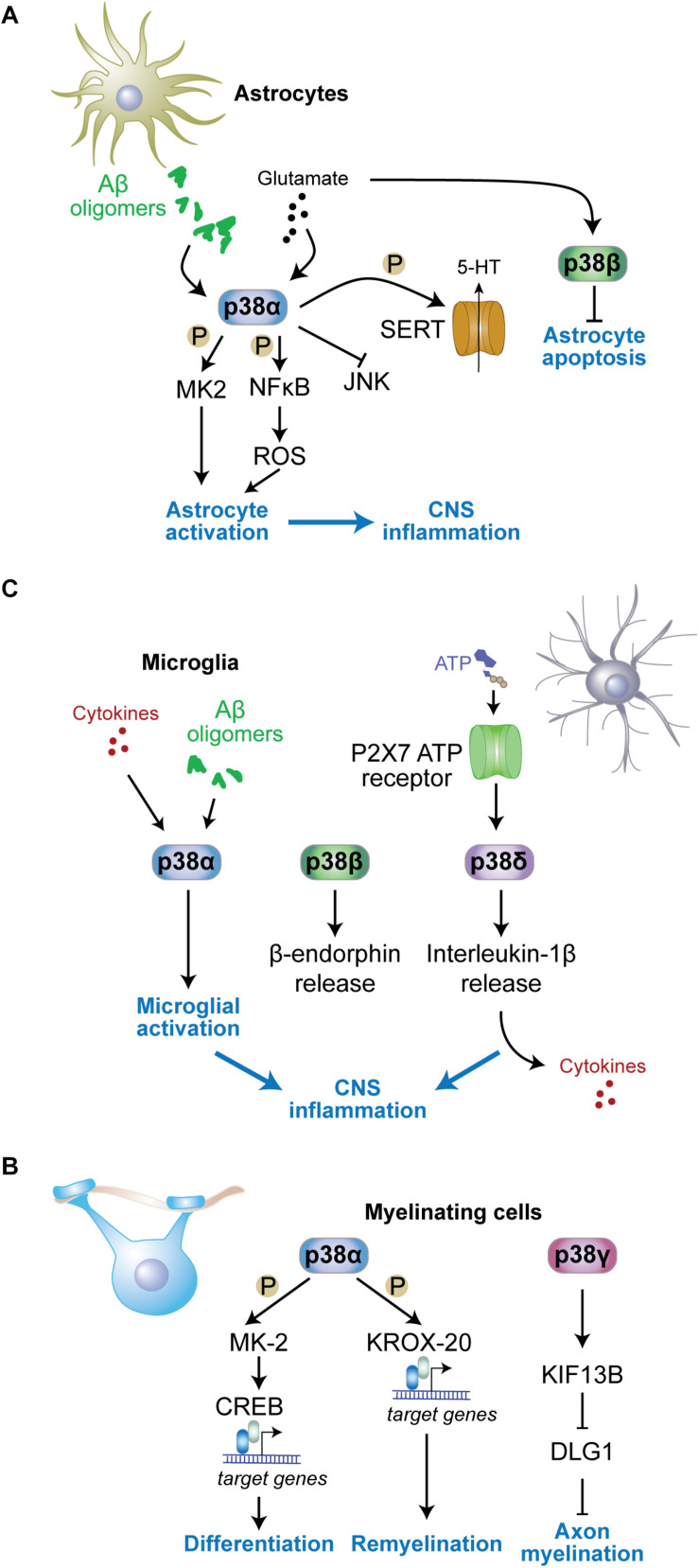
Functions of p38 kinases in non-neuronal cells. **(A)** In astrocytes, p38α is activated by stimuli including amyloid-β oligomers or excess glutamate and contributes to astrocyte activation and neuroinflammation. MK2 is activated in astrocytes downstream of p38α. p38α activates NFκB resulting in reactive oxygen species (ROS) production and astrocyte activation. Astrocyte p38α inhibits c-jun N-terminal kinase (JNK) activity, and enhances serotonin (5-HT) uptake into astrocytes through phosphorylation of serotonin transporter (SERT). p38β is also activated by glutamate in astrocytes yet contributes to astrocyte survival by inhibiting apoptosis. **(B)** In myelinating cells, p38α promotes remyelination through phosphorylation of key myelin transcription factor KROX-20 and downstream gene expression. p38γ contributes to diameters of myelin sheaths by reducing levels of disks large homolog 1 (DLG1), a inhibitor of myelination, through control of KIF13B levels. **(C)** In microglia, p38α is activated by stimuli including cytokines and amyloid-β oligomers, while microglial p38δ is activated downstream of P2 × 7 adenosine triphosphate (ATP) receptors. Both active p38α and p38δ contribute to neuroinflammation by promoting further cytokine release from microglia. p38β promotes release of microglial β-endorphin.

p38α regulates astrocytic neuroinflammatory responses during brain injury or neurotoxic insults (Figure 3A). Astrocytic expression of p38α increases upon excitotoxic stress using kainic acid (Che et al., 2001b). Both inhibitor studies and genetic ablation of *p38*α suggest that p38α promotes cytokine and chemokine production during CNS inflammation (Lee Y.B. et al., 2000; Lee et al., 2003; Lo et al., 2014). Deletion of *p38*α in astrocytes using a *Gfap2*-driven cre deleter mouse strain resulted in an altered immune response in the brain and modulated the activation state of astrocytes (Lo et al., 2014). Interestingly, JNK activation in *p38*α-deficient astrocytes was enhanced (Lo et al., 2014). This is consistent with reports of JNK inhibition by p38α in other cell types including hepatocytes and neurons (Hui et al., 2007; Heinrichsdorff et al., 2008; Stefanoska et al., 2018). Furthermore, p38α enhances NFκB and MK2 signaling as well as production of reactive oxygen species in astrocytes (Nahirnyj et al., 2013; Lo et al., 2014), which are required for cytokine production downstream of p38α in astrocytes (Lo et al., 2014) and other cell types (Bachstetter et al., 2013). Interestingly, deletion of *p38*α in astrocytes resulted in fewer activated astrocytes, yet increased influx of activated microglia and peripheral immune cells into sites of CNS inflammation (Lo et al., 2014), suggesting that astrocytic p38α regulates recruitment of glial and immune cells, likely through regulation of chemokine release. Consistent with differential roles of p38α in astrocytes and microglia, p38α can affect mounting and resolution of peripheral inflammatory processes differently through its action in different cell types (Kim et al., 2008).

p38α was shown to regulate serotonin metabolism in serotonergic neurons, impacting on anxiety and addiction behavior (Bruchas et al., 2011; Ehrich et al., 2015). This function of p38α in 5HT neurons is enhanced by astrocytic p38α, which regulates serotonin uptake into astrocytes (Bruchas et al., 2011). Future studies may want to address whether p38α regulates uptake and metabolism of other neurotransmitters in astrocytes.

p38β regulates cell survival of astrocytes by protecting them from apoptosis (Shin et al., 2011). Kainic acid induces different kinetics of expression of p38β compared with p38α in astrocytes based on immunoreactivity (Che et al., 2001a). The protective function of p38β in astrocytes may be related to this difference in expression, yet detailed signaling events involved in this function of p38β are unknown.

## Microglial Functions of p38 Kinases

Microglia express all four p38 kinases, and microglial p38 kinases have partially overlapping as well as redundant functions (Figure 3B). Inhibitor and gene knockout studies imply p38 and downstream pathways in mediating neuroinflammation in multiple contexts and models.

p38 kinases regulate microglial activation upon exposure to proinflammatory cytokines and amyloid-β or during tissue injury (Bachstetter et al., 2013; Swaroop et al., 2016). Microglia-specific deletion of *p38*α (using *Cx3cr1*-cre delete) reduces inflammatory reaction, cytokine levels, and microglial recruitment to damaged brain regions in a mouse model of traumatic brain injury (Morganti et al., 2019). Results from lysozyme promoter-driven *p38*α deletion in myeloid cells that partially contribute to microglia populations resulted, however, in increased levels of cytokines during acute injury-related inflammation (Bachstetter et al., 2013). Cytokine production from microglia is regulated downstream of p38α by MK2 (Culbert et al., 2006; Ghasemlou et al., 2010). Thus, p38α and downstream pathways have an established role in microglial cytokine production. p38α may indeed be a key p38 kinase in microglial activation. A study suggested p38β is not involved in microglia activation (Culbert et al., 2006; Xing et al., 2013).

p38α regulates additional aspects of microglial biology. p38α inhibits autophagy in microglia by phosphorylation of ULK1 (He et al., 2018). Autophagy in microglia was shown to inhibit inflammatory response (Su et al., 2016; Bussi et al., 2017). In these experiments, microglia lacking p38α were found to release more cytokines (He et al., 2018), similar to acute inflammatory response in p38α-deficient microglial cells (Bachstetter et al., 2013).

Microglia release exosomes (Potolicchio et al., 2005; Andaloussi et al., 2013), a mechanism that is important in brain physiology and for spreading of pathology in neurodegenerative diseases (Jan et al., 2017). Release of microparticles and IL-1β from microglia downstream of sphingomyelinase activity was shown to be p38-dependent based on an inhibitor that targets p38α and p38β (Bianco et al., 2009). SB203580, the inhibitor used in this work, has been, however, shown to affect autophagy (Menon et al., 2015) and therefore may affect vesicle trafficking and release (Tian et al., 2019). Thus, studies in *p38* knockout microglia are needed to clarify the role of p38 kinases.

p38 kinases can regulate neuroprotective pathways through their action in microglia. The neuroprotective biacetophenone cynandione A was shown to induce microglial β-endorphin expression, which was blocked by knockdown of p38β (Huang et al., 2017). Microglia-derived β-endorphin dampens nociceptive stimuli through a mechanism that involves glucagon-like peptide-1 receptors (Gong et al., 2014). These results suggest that p38β has a function in modulation of nociceptive sensory systems (Huang et al., 2017).

p38 kinases in microglia may have isoform-specific functions downstream of different receptors. p38β acts downstream of glucagon-like peptide-1 receptors (Gong et al., 2014). p38δ was shown to act downstream of the ATP receptors P2 × 7 in microglia, enhancing the release of IL-1β and inflammation within the spinal cord (Clark et al., 2010). It will be interesting to explore interaction partners and engagement in distinct signaling pathways for different p38 kinases downstream of microglial surface receptors. Molecular determinants within the primary sequence of the four p38 kinases that would support mechanistic engagement with specific receptor complexes are unknown. An exception is p38γ, which carries a C-terminal PDZ interaction motif to recruit p38γ into complexes in neurons and other cells based on interaction with cognate PDZ domain-containing proteins (Sabio et al., 2005; Ittner et al., 2016).

## Functions of p38 Kinases in Myelinating Cells

p38 kinases regulate aspects of myelinating cell function (Figure 3C). Differentiation of oligodendrocytes *in vivo* is controlled by the p38α-MK2 pathway (Fragoso et al., 2007; Haines et al., 2010) through downstream effects on transcription factor cAMP response element binding protein (CREB) (Bhat et al., 2007). However, genetic deletion of *p38*α in oligodendroglia, using the *NG2* cre delete strain, resulted in no gross myelination deficits, albeit delayed oligodendrocyte differentiation, alterations in the ultrastructure of myelin, and delayed myelination in the corpus callosum (Chung et al., 2015). Deficiency in oligodendroglial *p38*α has no effect on myelination when inducing demyelination with LPS or oxidative stress (Chung et al., 2015) yet enhances remyelination after cuprizone-induced demyelination (Chung et al., 2015). Consistent with these results genetic heterozygosity in *p38*α improves recovery in an animal model of spinal cord injury (Umezawa et al., 2017). This function of p38α is relevant to the peripheral nervous system as well. Mechanistic studies in Schwann cells with genetic deletion of *p38*α support that p38α limits myelination and showed accelerated myelination by *p38*α-deficient Schwann cells postnatally (Roberts et al., 2017). This function of p38α is partially mediated through transcriptional control of Krox-20 (Hossain et al., 2012), a master transcription factor in Schwann cells.

Functions of p38 kinases other than p38α in myelinating cells are less well characterized. The homolog of the disks large Dlg1, a MAGUK family member that has an inhibitory function in myelination of the peripheral system (Bolis et al., 2009), undergoes post-translational modification downstream of Kif13b, a kinesin-like factor regulating intracellular localization of MAGUKs (Yoshimura et al., 2010). Dlg1 forms a complex with KIF13B and p38γ, is then phosphorylated by p38γ and subsequently ubiquitinated. These modifications in response to KIF13B activity reduce cellular levels of Dlg1 and result in enhanced myelinating function of Schwann cells (Noseda et al., 2016). Addressing sciatic nerve myelination, this study found reduced myelin sheath thickness in *p38*γ knockout nerves, yet normal axonal numbers and fiber diameter (Noseda et al., 2016). Whether the same mechanism regulates myelinating cells of the central nervous system remains to be determined. Nevertheless, the impact of *p38*γ on Dlg1 in CNS myelination processes may be limited to specific stimuli and/or non-physiological conditions because global knockout of *p38*γ does not result in gross myelination defects in white matter tracks of mice (Ittner et al., 2016; Noseda et al., 2016). The p38γ – Dlg1 pathway may potentially have different functions in oligodendrocytes as Dlg1 acts to promote myelinating function of cells through a pathway involving PTEN-dependent inhibition of Akt (v-AKT murine thymoma viral oncogene homolog) activity (Noseda et al., 2016; Hughes and Appel, 2019). Thus, p38 kinase function in myelinating cells of the peripheral and central nervous system differ.

## p38 Kinases in Other CNS Cell Types

p38 kinases are involved in functions of additional cell types present in the CNS, including endothelial cells and pericytes, and contribute to neuroinflammation and control of the cerebrovasculature and blood–brain-barrier (BBB). p38 activation promotes experimental BBB disruption, which is inhibited by TGF-β-induced expression of MKP1 (Tong and Hamel, 2007). TGF-β1 and p38 MAP kinase reciprocally regulate pericyte expression of MMP-9 (Takahashi et al., 2014). This suggests p38 signaling pathway involvement in regulation of neurovascular homeostasis under conditions of stress exposure.

## Roles of p38 Kinases in Cognitive Functions

p38 is activated by stress stimuli in cells (Raingeaud et al., 1995). Deletion of *p38* genes – with the exception of *p38*α – does not result in obvious changes in cognition or behavior of mice under resting conditions (Beardmore et al., 2005; del Barco Barrantes et al., 2011; Ittner et al., 2016). Even ablation of *p38*α in neurons, astrocytes or microglia does not result in apparent basal changes (Bruchas et al., 2011; Ittner et al., 2016; Colie et al., 2017; Stefanoska et al., 2018). Several key functions of p38 kinases in cognition became evident with challenge from drugs, cognitive tasks or psychogenic stress.

### Anxiety

**FIGURE 4:**
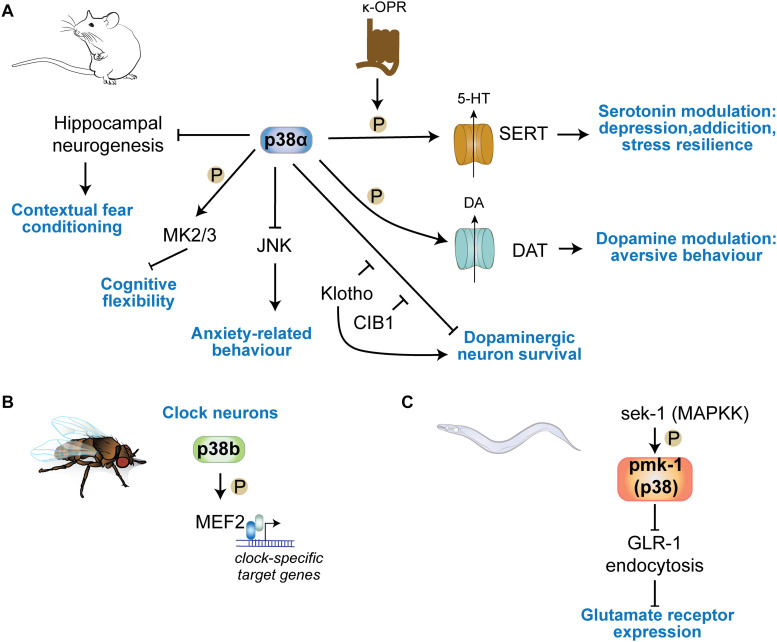
p38 kinases in physiologic central nervous functions. **(A)** p38α regulates learning in contextual fear conditioning through regulation of hippocampal neurogenesis. Neuronal p38α inhibits JNK activation in neurons to modulate anxiety-related behavior. p38α downstream of κ-opioid channels regulates serotonin reuptake in serotonergic cells by direct phosphorylation of SERT. This mechanism is involved in serotonin-regulated aspects of depression, addiction, social defeat stress. DA re-uptake by phosphorylation of DAT in DA neurons. **(B)** p38b regulates circadian rhythms in *Drosophila* by transcriptional regulation of clock neuron gene expression through the transcription factor MEF2C downstream of p38b. **(C)** The sek1 pmk1(p38) pathway in *C. elegans* regulates glutamate receptor GLR-1 trafficking at neuronal synapses.

*p38*α deletion in neurons results in increased anxiety tested by open field paradigm and elevated plus maze (Figure 4A) (Stefanoska et al., 2018). Grehlin, a gastric peptide with anxiolytic activity in the brain, was shown to require p38 activity based on inhibitor injection into hippocampus (Han et al., 2019). p38α in serotonergic cells of the Raphe nucleus is necessary for SERT membrane expression and 5HT re-uptake induced by chemical/environmental stressors, reducing serotonin-modulated behaviors (Bruchas et al., 2011; Baganz et al., 2015). Activated p38α in presynaptic VTA neurons phosphorylate GIRK Kir3.1 to decrease somatic excitability and exocytosis of dopamine, allowing for development of place aversion in a behavioral conditioning task (Ehrich et al., 2015). Anxiety-related behavioral phenotypes are not apparent in p38γ knockout mice (Pogozelski et al., 2009; Ittner et al., 2016; Noseda et al., 2016) and have not been addressed for in p38β or p38δ knockout mice (Beardmore et al., 2005; Sumara et al., 2009).

### Addiction Behavior

p38 regulates drug seeking behavior, based on mouse models of addiction (Figure 4A). Cocaine application activated p38 in nucleus accumbens and prefrontal cortex, and results in recruitment of active p38 to norepinephrine transporter (NET) and DA transporter (DAT) (Mannangatti et al., 2015). p38 can phosphorylate NET at Thr-30 and blocking this interaction in mice reduced cocaine-conditioned place preference in mice. Furthermore, morphine-conditioned place preference was not affected by p38 inhibition, suggesting a cocaine-specific engagement of p38 in this behavior (Mannangatti et al., 2015). Interestingly, this study suggests that amphetamine – a drug of addiction that targets the serotonergic system (Kirby et al., 2011; Steinkellner et al., 2015) – selectively induces p38 association with SERT (Mannangatti et al., 2015), confirming previous findings of regulation of SERT and addiction behavior mediated through serotonergic neurons (Bruchas et al., 2007; Bruchas et al., 2011). κ-opioid receptors regulate drug reinstatement in rat models of cocaine self-administration (Beardsley et al., 2005; Graziane et al., 2013; Ehrich et al., 2015; Heinsbroek et al., 2018). This action of κ-opioid receptor signaling is dependent on p38 (Heinsbroek et al., 2018). Ablation of *p38*α serotonergic neurons or astrocytes lower reinstatement of cocaine preference (Bruchas et al., 2011), providing genetic evidence for a significant role of p38 – and in particular p38α – in addiction.

### Learning and Memory

Memory tests in neuron-specific *p38*α knockout mice revealed no deficits in memory acquisition or retrieval at 6–8 months of age (Stefanoska et al., 2018) (Figure 4A). Contextual fear condition DN p38α knock-in, in which the activation motif is mutated to Ala-Gly-Phe, mice show enhanced performance (Cortez et al., 2017). DN p38α mice were significantly protected from age-dependent reduction in hippocampal adult neurogenesis (Cortez et al., 2017). This suggests that p38α regulates aspects of memory function through inhibiting neurogenic precursor function. Thus, evidence suggest that p38 kinases have limited impact on memory acquisition. However, downstream substrates of p38α/β, MAPKAP kinases 2 and 3, are important for AMPAR endocytosis during long-term depression and regulate cognitive flexibility in spatial memory tasks (Eales et al., 2014). Other p38 kinases such as p38γ or p38δ appear dispensable for physiologic memory function. Knocking out p38γ or increasing activity of neuronal p38γ results in not alteration of memory function in mice (Ittner et al., 2016), even at advanced age (*unpublished results*).

## p38 Kinases in CNS Disorders and Neurodegeneration

p38 kinases have been linked to several neurological disorders, in particular, when associated with neuroinflammatory responses. This is largely based on their prominent role in cytokine production and release from immune cells including microglia (Lee Y.B. et al., 2000). Despite this role in neuroinflammation, distinct involvement of individual p38 kinases in pathways in different types or even sub-types of cells of the nervous system make contributions to disease processes. This is either through physiologic, protective mechanisms that dampen disease development or by enhancing disease progression through promoting toxic signaling.

### Ischemia

**FIGURE 5:**
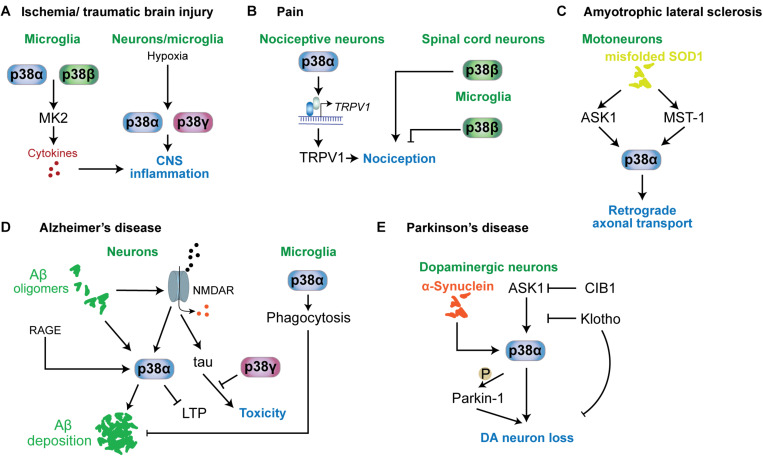
p38 functions in neurological and psychiatric disorders. Mechanisms involving p38 kinases have been described for a variety of neurological and psychiatric disorders. **(A)** In ischemia or traumatic brain injury, microglial p38α and p38β contribute to neuroinflammation by targeting MAPKAP kinase-2 (MK2), which promotes the release of proinflammatory cytokines. This mechanism is relevant to other conditions associated with neuroinflammation. Hypoxia is a key stimulus activating p38α and p38γ in neurons and microglia. **(B)** In nociception and pain, p38α in nociceptive neurons promotes the expression of TRPV1 channels that contribute to nociception. p38β in spinal cord neurons promotes nociception, while microglial p38β has an inhibitory role in nociception. **(C)** In ALS mouse models based on mutant SOD1, p38α downstream of ASK1 or MST1 is activated and impairs retrograde transport. **(D)** In Alzheimer’s disease, neuronal p38α can be activated by a variety of stimuli, including amyloid-beta, RAGE or aberrant glutamate receptor engagement. p38α activity promotes amyloid aggregation, LTP and neuroinflammation. p38γ inhibits the toxic function of tau in neurons by direct phosphorylation of tau. Interestingly, microglial p38α reduces amyloid plaque load in models of AD by promoting phagocytic activity. **(E)** In Parkinson’s disease, p38α in dopaminergic neurons is activated by alpha-synuclein aggregates. p38α promotes DA neuron loss by direct phosphorylation of Parkin-1. Activity in DA neurons is regulated by ASK1 upstream of p38α. This pathway can be inhibited by survival factors CIB1 and klotho.

Ischemic conditions are associated with multiple neurological disorders, including stroke, brain injury (Castillo et al., 2004; Shichita et al., 2014) and involves multiple secondary responses such as hypoxic response, neuroinflammation and excitotoxicity (Chamorro et al., 2016). p38 becomes activated by hypoxic conditions in brain tissue (Bu et al., 2007; Lu et al., 2011; Li et al., 2013). Involvement in ischemia-induced neurologic disorder has been largely addressed by inhibitor studies (Barone et al., 2001; Legos et al., 2001; Piao et al., 2003). Inhibition of p38α and p38β in ischemic conditions lowers neuroinflammation and cytokine release (Barone et al., 2001; Legos et al., 2001; Piao et al., 2003; Sanchez et al., 2012) (Figure 5A). Work in cultured cells showed that p38α and p38γ are activated by hypoxia rather than p38β or p38δ (Conrad et al., 1999) and p38α is required for hypoxia-induced factor-1 (HIF-1) stabilization (Emerling et al., 2005). Whether p38α and/or p38γ are critical for hypoxic response in CNS cells remains to be determined, yet it may suggest that p38α and p38γ could be specific targets for treatment of brain ischemia. Understanding the function of these 2 kinases in different brain cell types will be required as hypoxia induces p38 activation in both neurons and microglia (Bu et al., 2007). Phosphatase STEP interacts with and reduces activation of p38, lowering neurotoxicity in ischemia models (Poddar et al., 2010; Deb et al., 2013). Promoting activity of STEP toward p38 is neuroprotective and reduces infarct size (Deb et al., 2013). Furthermore, p38α in astrocytes is responsive to hypoxia and astrocyte-specific deletion of p38α results in lower inflammatory responses, astrocyte recruitment and activation in a model of ischemic stroke (Roy Choudhury et al., 2014).

### Traumatic Brain Injury

p38α regulates neuroinflammation during traumatic brain injury (Figure 5A). Microglia-specific deletion of *p38*α protects mice in a model of traumatic brain injury or LPS-driven neuroinflammation, lowering activation of microglia, influx of peripheral inflammatory cells, synaptic loss and ameliorates motor impairments (Bachstetter et al., 2013; Morganti et al., 2019). Both the involvement of p38 (foremost p38α) in neuroinflammation during TBI is largely based on the function of the p38α-MK2 pathway in cytokine production (Bachstetter et al., 2011, 2013; Morganti et al., 2019). Consistent with the prominent role of p38α in microglia in promoting TBI, inhibition of p38α and p38β had no effect on injury in a neuronal culture model (Mori et al., 2002). However, this early study did not find an effect of p38 inhibition by p38α/β-specific inhibitor SB203580 on the lesion size or motor performance after cerebrocortical injury (Mori et al., 2002). These results indicate cell-type specific contributions of p38 to pathomechanisms in TBI.

### Pain

p38 kinases are involved in nociception (Crown et al., 2008) and neuropathic pain (Svensson et al., 2003; Svensson et al., 2005) (Figure 5B). Antinociceptive action was found in animal models of pain for p38α inhibitors (Sweitzer et al., 2004; Galan-Arriero et al., 2015). Mechanism of action of p38α in nociception are based on regulation of nociceptive channels. Hyperalgesia maintained by p38-mediated TRPV1 levels in sensory neurons (Ji et al., 2002). Other p38 kinases have different functions in nociception than p38α. Knockdown of p38β within the spinal cord of rats induced hyperalgesia upon spinal cord injury (Svensson et al., 2005). Thus roles of p38α in neurons and for p38β in microglia promote hyperalgesia (Svensson et al., 2005; Fitzsimmons et al., 2010). Other work, however, suggested no involvement of p38β in microglial activation (Xing et al., 2013) or even a protective effect of microglial p38β from neuropathic pain (Huang et al., 2017). Taken together, mechanisms involving p38 kinases in nociception are not extensively understood and may depend on the type and time course of pain stimulus. However, overall evidence supports p38 as drug targets in treatment of pain.

### Amyotrophic Lateral Sclerosis (ALS)

Amyotrophic lateral sclerosis, also known as Lou Gehrig’s disease, is characterized by specific neurodegeneration within spinal cord, brain stem and cerebrum (Karitzky and Ludolph, 2001; Hammad et al., 2007). Increased activity of stress-activated MAP kinase was detected in cortical neurons of an ALS mouse model (Holasek et al., 2005) while higher p-p38 levels have been detected in human post-mortem ALS brains (Krieger et al., 2003; Sama et al., 2017) and mouse models of ALS (Tortarolo et al., 2003; Holasek et al., 2005; Veglianese et al., 2006; Sama et al., 2017). Activation of p38 in ALS models is associated with activated upstream kinase ASK1 and markers of apoptosis at an early stage (Wengenack et al., 2004) and mammalian sterile-20-like kinase 1 was shown to activate p38 in a SOD1 mouse model (Lee et al., 2013) (Figure 5C). SOD1 mutations are prevalent in ALS (Sau et al., 2007; Farrawell et al., 2015). Inhibition of p38 has protected mutant SOD1 transgenic mice from motor neuron loss (Dewil et al., 2007). Pathogenic SOD1 inhibits axonal transport in motor neurons through activation of p38α downstream of ASK1 (Morfini et al., 2013; Song et al., 2013). Consistently, p38α inhibition restores retrograde axonal transport in a mouse model of ALS based on expression of mutant SOD1 (Gibbs et al., 2018). Thus, p38 MAP kinase - in particular p38α – is a promising therapeutic target in SOD1-associated forms of ALS.

Familial ALS can be associated with other mutations than in *SOD1* (Farrawell et al., 2015). FUS (fused in osteosarcoma) mutations are linked to familial forms of ALS (Vance et al., 2009). Brain tissue from ALS with FUS mutations displayed increased levels of activated p38 (Sama et al., 2017) and mutant FUS affects axonal transport through p38 activity (Sama et al., 2017). These results suggest that p38 activation and downstream effects are a common mechanism in multiple forms of ALS pathogenesis.

### Alzheimer’s Disease (AD)

Alzheimer’s disease is the most prevalent form of neurodegenerative dementia and associated with extracellular plaques derived from amyloid-β peptide aggregates and intracellular neurofibrillary tangles, which consist mainly of hyperphosphorylated forms of the neuronal tau protein (Ittner and Gotz, 2011; Jin et al., 2011; Bloom, 2014; Ittner and Ittner, 2018). Hence, much research has focussed on amyloid-β (Aβ) and tau as central factors in the diseases (Ittner and Gotz, 2011; Jin et al., 2011; Bloom, 2014).

Increased levels of activated p38 has been found in neurons from AD brains (Sun et al., 2003) and tissue samples from transgenic AD mouse models based on increased expression of pathogenic amyloid-β precursor protein (APP) (Ittner et al., 2014; Criscuolo et al., 2017) (Figure 5D). As such, p38 MAP kinase has been suggested as drug target for AD treatment (Munoz and Ammit, 2010). Aβ can activate p38 in neurons (Origlia et al., 2008; Li S. et al., 2011), which may be mediated through NMDA receptors because NMDAR blocker MK801 suppressed p38 activation in APP transgenic mice (Ittner et al., 2014). Furthermore, p38α – was shown to mediate some of the neurotoxic effects of Aβ, including suppression of long-term potentiation and neuronal death in culture based both on pharmacological inhibition (Li S. et al., 2011) and neuron-specific deletion of *p38*α (Colie et al., 2017). The latter work also found a reduction in Aβ deposition in AD mice crossed with neuron-specific *p38*α knockout mice resulting in lower amyloid plaque burden (Colie et al., 2017). Thus, p38α appears to regulate both toxic signals downstream of Aβ in neurons as well as Aβ processing and/or deposition. p38α affects Aβ processing through regulation of protein levels of the APP-processing protease BACE1 in neurons (Schnoder et al., 2016) and through modulation of autophagy (Alam and Scheper, 2016), both of which increase the amount of extracellular Aβ. Results from p38α inhibition in microglia, however, suggest that p38α promotes phagocytosis of amyloid-beta by microglia (Reed-Geaghan et al., 2009). p38α inhibition also reduces tau pathology, i.e., levels of hyperphosphorylated tau, through increasing autophagy by microglial (Maphis et al., 2016). Thus, p38α may serve several functions in the context of amyloid and tau pathology. Nevertheless, p38α has been put forward as key drug target in amyloidosis and associated dementia. Experiments with an p38α-specific inhibitor in AD mouse models suggest that this strategy is sufficient to ameliorate Aβ-induced cognitive impairment (Alam and Scheper, 2016; Alam et al., 2017).

Receptor for advanced-glycation end products (RAGE) was shown to activate p38, resulting in LTP inihibition (Origlia et al., 2008). Signals through RAGE and p38 may converge on LTP inhibition in AD. Antagonizing RAGE by a dominant negative RAGE receptor in a mouse model of AD prevents p38 activation in the hippocampus, LTP and memory impairment (Criscuolo et al., 2017).

p38γ MAP kinase has recently been shown to mediate a neuroprotective function in AD mouse models. p38γ differs from other p38 kinases in harboring a C-terminal PDZ interaction motif, which mediates its localization to the post-synapse (Sabio et al., 2005; Ittner et al., 2016). Aβ had previously been shown to mediate its toxic effects through tau (Roberson et al., 2007; Ittner et al., 2010), laying the foundation to the concept of the tau-dependent ‘amyloid cascade,’ which links both key factors in a central toxic signaling pathway (Ittner and Gotz, 2011). Mechanistically, tau, a predominantly axonal protein in neurons (Harada et al., 1994), is partially localizing to dendrites where it interacts with post-synaptic scaffolding factor PSD-95 downstream of NMDA receptors (Ittner et al., 2010). This interaction is required to mediate toxic effects of Aβ in neurons and in mice (Ittner and Ittner, 2018). p38γ interacts with PSD-95 and was shown to phosphorylate tau predominantly on Threonine-205 in this complex. This phosphorylation event results in disruption of the PSD-95-tau complex and inhibits toxic signals of Aβ (Ittner et al., 2016). Thus, this mechanism involving post-synaptic p38γ showed a novel neuroprotective function for this p38 kinase and revealed an unprecedented function of tau phosphorylation in inhibiting a pivotal toxicity mechanism of Aβ (Ittner and Ittner, 2018; Ittner et al., 2020). Interestingly, neither p38α, p38β nor p38δ were able to mediate the same neuroprotective tau phosphorylation and inhibition of Aβ toxicity as p38γ (Ittner et al., 2016), likely due to the distinct localization of p38γ in neurons at post-synapses (Sabio et al., 2005; Ittner et al., 2016).

### Parkinson’s Disease (PD)

Loss of dopaminergic (DA) neurons is a hallmark of PD (Antony et al., 2013). p38 activity was found in DA neurons in the substantia nigra pars compacta in PD patient samples and PD mouse models (Ferrer et al., 2001; Karunakaran et al., 2008; Ray et al., 2015) (Figure 5E). Activation of p38 induced nuclear localization of p53 in DA neurons in a MPTP model whereas inhibition of p38 protects DA neurons (Karunakaran et al., 2008). In addition, p38 controls DA neuron survival and resistance to oxidative and inflammatory stress (Jeohn et al., 2002; Brobey et al., 2015a, b). p38 activity downstream of ASK1 is inhibited by klotho (Brobey et al., 2015b), a factor that prevents premature aging and striatal DA neuron loss (Kosakai et al., 2011). A transgenic PD mouse model based on mutant α-synuclein expression presented with activation of p38, which phosphorylates Parkin-1 and promotes mitochondrial dysfunction and DA neuron loss (Chen et al., 2018) (Figure 5E).

Though loss of DA neurons is the most prominent feature, neuronal dysfunction and degermation is not strictly limited to DA neurons. Serotonergic loss contributes to symptoms and neuropsychiatric changes in PD (Politis and Niccolini, 2015; Maillet et al., 2016; Schindlbeck and Eidelberg, 2018). This is particularly true for PD associated with mutations in the α-synuclein-encoding *SNCA* gene (Wilson et al., 2019). Bruchas et al. (2011) indicate that p38α regulates serotonin release and reuptake through serotonin transporter cell surface trafficking (Ehrich et al., 2015). This function of p38α may be important in serotonergic dysfunction in PD, making p38α a potential drug target for serotonin-associated symptoms of PD.

### Huntington’s Disease

Huntingtin (Htt) poly-glutamine expansion is a hallmark of Huntington’s disease (Scherzinger et al., 1997). Activated p38 was found in multiple HD models (Gianfriddo et al., 2004; Rangone et al., 2004; Wang et al., 2010). Poly-glutamine Htt activates p38 MAPK kinase (Taylor et al., 2013), yet conflicting evidence suggests mutant Htt activates ERK MAP kinase rather than JNK or p38 MAP kinases (Apostol et al., 2006). However, MAP kinase phosphatase-1 (MKP-1), which specifically deactivates p38 and JNK has a protective function in striatal neurons of a Huntington’s model (Taylor et al., 2013), suggesting that activity of p38 contributes to toxic signals induced by mutant Htt. Consistently, inhibition of p38α and p38β protects from neurotoxicity induced by polyglutamic (Tsirigotis et al., 2008).

### Spinal Cord Injury (SCI)

p38α plays a central role in inflammatory process in SCI through translation and stabilization of inflammatory mRNAs. For example, MAPKAPK2 or MK2, the downstream kinase of p38, interferes with the interaction between the binding factors and AU-rich elements (AREs) which in turn stabilizes TNF-α mRNA [reviewed in Dean et al. (2004)]. In addition, p38/MK2-induced mRNA stabilization also controls the expression of IL-6 which triggers activation and infiltration of leukocytes in SCI (Zhao et al., 2008). Thus, p38α can initiate major SCI-related proinflammatory cytokines in the post-traumatic inflammation. Extensive research utilizing various p38α inhibitors and genetically modified mice that p38α plays an important role in various stages of inflammatory process [reviewed in Kasuya et al. (2018)]. Increased spatial activation of p38α was observed in neurons, oligodendrocytes, microglia/macrophages, infiltrated neutrophils, and reactive astrocytes which formed a glial scar following SCI [reviewed in Kasuya et al. (2018)]. Activation of p38α in activated microglia/macrophages in SCI models induces iNOS expression, contributing to loss of neuronal cells (Xu et al., 2006). IL-1β and p38 phosphorylation was detectable prior to neuronal apoptosis, and IL-1 receptor antagonist and p38 inhibition, suppressed p38 phosphorylation and neuronal apoptosis, indicating a critical involvement of IL-1β/p38α signaling axis in neuronal apoptosis post-SCI (Wang et al., 2005).

## Conclusion and Future Questions

The last two decades of research have seen p38 kinases move from stress-activated kinases to signal transducers in multiple physiologic and pathophysiologic functions. This is particularly true for cells of the central nervous system, where specific functions of p38 kinases were revealed in all major cell types contributing to brain physiology, cognition and behavior as well as disease processes. These discoveries are attributable to the development of more specific genetic and pharmacologic tools that are able to dissect functions of individual kinases. Development of these tools has made answering the following outstanding questions on p38 in the CNS feasible:

• What is the brain region- and cell type-specific expression of individual p38 kinases?
• In tissue with co-expression of p38 isoforms, are the kinases functionally distinct or is there redundancy?
• Does p38α regulate broadly regulate biogenic amine neurotransmission through parallel mechanisms? Can these mechanisms be targeted to treat disorders associated with specific neurotransmitter systems?
• What distinct roles do p38β, p38γ and p38δ have in behavior and cognition?
• What is the therapeutic potential of p38α inhibition for treatment of neurodegeneration, addiction, and anxiety? What are the mechanisms of action? Could drugs target the role of p38α in neuroinflammation, autophagy, regulating synaptic plasticity, mediating axonal transport?
• What is the therapeutic potential of p38γ for conditions of excitotoxicity and neurodegeneration?
• What is the therapeutic potential of p38β in motor neuron disease/ALS targeting its function in microglia?

Investigating mechanisms involving p38 kinases provides new molecular insights into brain physiology and opens new therapeutic avenues to understand and target neurological disorders. From the basis of roles in inflammation by initial discoveries of p38, a more diversified picture of p38 kinase functions is still emerging.

## Author Contributions

AI, PA, EP, and KS wrote first draft of the manuscript. PA edited sections of cell-type specific functions of p38 and contributed to preparation of figures. EP edited sections of MAP kinase signaling cascade and prepared figures. KS wrote section of disease-related functions of p38 in CNS. AT and HA edited the manuscript draft and contributed to figure preparation. AI designed concept and outline of the manuscript. All authors contributed to the article and approved the submitted version.

## Conflict of Interest

The authors declare that the research was conducted in the absence of any commercial or financial relationships that could be construed as a potential conflict of interest.
